# Differential Transcriptomics Analysis of IPEC-J2 Cells Single or Coinfected With Porcine Epidemic Diarrhea Virus and Transmissible Gastroenteritis Virus

**DOI:** 10.3389/fimmu.2022.844657

**Published:** 2022-03-25

**Authors:** Lina Song, Jing Chen, Pengfei Hao, Yuhang Jiang, Wang Xu, Letian Li, Si Chen, Zihan Gao, Ningyi Jin, Linzhu Ren, Chang Li

**Affiliations:** ^1^ College of Veterinary Medicine, Key Lab for Zoonoses Research, Ministry of Education, Jilin University, Changchun, China; ^2^ Research Unit of Key Technologies for Prevention and Control of Virus Zoonoses, Chinese Academy of Medical Sciences, Changchun Institute of Veterinary Medicine, Chinese Academy of Agricultural Sciences, Changchun, China; ^3^ College of Animal Sciences, Jilin University, Changchun, China

**Keywords:** porcine epidemic diarrhea virus (PEDV), transmissible gastroenteritis virus (TGEV), differential transcriptomics, coinfection, interferon-stimulated genes (ISGs), interferon-induced transmembrane protein (IFITM)

## Abstract

Porcine epidemic diarrhea (PED) and transmissible gastroenteritis (TGE) caused by porcine epidemic diarrhea virus (PEDV) and transmissible gastroenteritis virus (TGEV) are two highly contagious intestinal diseases in the swine industry worldwide. Notably, coinfection of TGEV and PEDV is common in piglets with diarrhea-related diseases. In this study, intestinal porcine epithelial cells (IPEC-J2) were single or coinfected with PEDV and/or TGEV, followed by the comparison of differentially expressed genes (DEGs), especially interferon-stimulated genes (ISGs), between different groups *via* transcriptomics analysis and real-time qPCR. The antiviral activity of swine interferon-induced transmembrane protein 3 (sIFITM3) on PEDV and TGEV infection was also evaluated. The results showed that DEGs can be detected in the cells infected with PEDV, TGEV, and PEDV+TGEV at 12, 24, and 48 hpi, and the number of DEGs was the highest at 24 hpi. The DEGs are mainly annotated to the GO terms of protein binding, immune system process, organelle part, and intracellular organelle part. Furthermore, 90 ISGs were upregulated during PEDV or TGEV infection, 27 of which were associated with antiviral activity, including ISG15, OASL, IFITM1, and IFITM3. Furthermore, sIFITM3 can significantly inhibit PEDV and TGEV infection in porcine IPEC-J2 cells and/or monkey Vero cells. Besides, sIFITM3 can also inhibit vesicular stomatitis virus (VSV) replication in Vero cells. These results indicate that sIFITM3 has broad-spectrum antiviral activity.

## Introduction

Porcine epidemic diarrhea (PED) and transmissible gastroenteritis (TGE) caused by porcine epidemic diarrhea virus (PEDV) and transmissible gastroenteritis virus (TGEV), respectively, are two highly contagious intestinal diseases in the swine industry worldwide, which are characterized by acute gastroenteritis, watery diarrhea, and vomiting in pigs of almost all ages. Both viruses belong to the family *Coronaviridae* and genus *Alphacoronavirus* (1), with a positive-sense single-stranded RNA genome of about 28 kb encoding at least six open reading frames (ORFs): ORF1a, ORF1b, spike (S), envelope (E), membrane (M), and nucleocapsid (N) (2, 3).

TGEV has been spread in pigs for decades, whereas PEDV is considered as a new coronavirus detected in pigs (2, 3), especially the highly virulent PEDV that has recently emerged and caused great losses worldwide. Furthermore, coinfection of TGEV and PEDV is common in piglets with diarrhea (4–7). During infection, TGEV mainly infects the small intestine by interacting with host receptor amino peptidase N (APN, also named as CD13), sialic acid, and/or other cofactors (1, 8, 9). PEDV can directly infect the villous intestinal epithelial cells of the small intestine or nasal epithelial cells followed by dissemination from the nasal cavity to the intestinal mucosa by binding with sialic acid and other receptors (1, 10, 11). However, whether porcine APN is a functional receptor for PEDV infection is still controversial (1). Moreover, TGEV can damage the barrier integrity of intestinal porcine epithelial cells (IPEC-J2) in the early stage of infection by downregulating proteins related to tight and adhesion junction, while PEDV impairs the integrity of the cellular epithelial barrier (12). Both viruses can also affect the remodeling of microfilaments in IPEC-J2 cells, and the coinfection of PEDV and TGEV can increase the damage of tight junction and the remodeling of microfilaments (12). Besides, TGEV or PEDV infection reduced NHE3 activity and Na^+^ uptake of IPEC-J2 cells, which may be associated with the imbalance of Na^+^ in intestinal tissues, thus resulting in diarrhea in the infected animals (13). The differentially expressed genes (DEGs) in IPEC-J2 cells infected with virulent PEDV virus are mainly related to autophagy and apoptosis, while the DEGs were strongly enriched in immune responses/inflammation in the avirulent PEDV group (14). TLR3 inhibited the replication of avirulent PEDV by increasing the IFIT2 expression (14). Notably, a recent investigation showed that coinfection of TEGV and PEDV leads to recombinant chimeric swine enteric coronavirus (SeCoV) in Italy, Germany, and Slovakia (15–18), which implies the urgency of prevention and control of virus-related diseases. It was reported that viral nucleocapsid from different porcine enteric coronaviruses can differentially modulate PEDV replication by competitively interacting with PEDV nucleocapsid (19). Nucleocapsid from porcine deltacoronavirus (PDCoV) can significantly decrease PEDV replication, while overexpression of the TGEV nucleocapsid enhanced the virus replication (19). These results indicate that coinfection of different enteric coronaviruses may have different results on virus infection and host responses. However, little is known about the cell responses, especially host immune responses, after single or coinfection of PEDV and TGEV.

The ability of the host to inhibit virus infection largely depends on the effectiveness of the antiviral innate immune response, which leads to the upregulation of interferon (IFN), followed by activation of signal transduction cascades, and thus leading to the induction of hundreds of interferon-stimulated genes (ISGs) (20, 21). ISGs work alone or cooperatively to achieve one or more cellular outcomes, including antiviral defense, antiproliferative activity, and stimulation of adaptive immunity (20, 21). However, although the specific antiviral functions of some ISGs have been characterized, the functions of other ISGs have yet to be determined. Moreover, Zhao et al. found that IFN-λ1 has a stronger ability to induce ISGs against PEDV infection than IFN-α (22). TGEV infection stimulates the JAK-STAT1 signaling pathway and ISG expressions (23). However, the expression of ISGs after the infection of PEDV and TGEV alone or together remains to be studied.

In the present study, porcine IPEC-J2 cells were single or coinfected with PEDV and/or TGEV, followed by the comparison of differentially expressed genes, especially ISGs, between different groups *via* transcriptomics analysis and real-time qPCR. The antiviral activity of interferon-induced transmembrane protein 3 (IFITM3) on PEDV and TGEV infection was also evaluated.

## Materials and Methods

### Cells and Viruses

IPEC-J2 cells (kindly provided by Dr. Shuqi Xiao), Vero E6 cells, and ST cells were maintained in Dulbecco’s modified Eagle medium (DMEM) (HyClone, Logan, UT, USA), supplemented with 10% fetal bovine serum (FBS, Gibco, Grand Island, NY, USA) and penicillin–streptomycin mixtures at 37°C and 5% CO_2_ atmosphere. Human lung epithelial (A549) cells, Baby hamster kidney cells (BHK-21), and chicken fibroblast cells (DF-1) were grown in complete DMEM with 10% fetal bovine serum (Gibco, USA) at 37°C in a 5% CO_2_ incubator (24, 25).

The PEDV strain (GenBank No.: OM814174) and the TGEV strain (GenBank No.: OM802899) were isolated in our laboratory previously. The PEDV was cultured in Vero cells supplemented with 5 μg/ml trypsin. Moreover, the TGEV was cultured in ST cells with DEME (2% FBS without penicillin–streptomycin). Vesicular Stomatitis Virus carrying green fluorescent protein gene (rVSV-GFP) was kindly provided by Professor Zhigao Bu as described previously (24, 25).

### Antibodies and Reagents

Mouse anti-PEDV N mAb (FITC) was purchased from Medgene Labs (Brookings, SD, USA). Mouse anti-PEDV S and Mouse anti-TGEV S polyclonal antibodies were prepared in our laboratory. Rabbit anti-IFITM3 polyclonal antibody was purchased from Proteintech (Wuhan, China). Rabbit anti-β-actin mAb was purchased from Cell Signaling Technology (Danvers, MA, USA). Lipofectamine 3000 Transfection Reagent and Lipofectamine™ RNAiMAX Transfection Reagent were purchased from Invitrogen (Carlsbad, CA, USA).

pLV-sIFITM3-Flag was constructed by our laboratory. Briefly, swine interferon-induced transmembrane protein 3 (sIFITM3) was amplified using primers sIFITM3-F and sIFITM3-R (**Supplemental Table 1**) and subcloned into pLV-EGFP (Inovogen Tech, Beijing, China) with *Eco*R I and *Xho* I, resulting in pLV-sIFITM3-Flag. Furthermore, sIFITM3 was also amplified using primers sIFITM3-F2 and sIFITM3-R2 (**Supplemental Table 1**), and subcloned into pCAGGS-Flag (Inovogen Tech, Beijing, China) with *Eco*R I and *Xho* I, resulting in pCAGGS-sIFITM3-Flag. The recombinant plasmids were verified by PCR and sequencing.

TRIzol reagent was purchased from Sangon Biotech (Shanghai, China). M-MLV Reverse Transcriptase RNase and GoTaq^®^ were purchased from Promega (Madison, WI, USA). HRP-labeled Goat Anti-Rabbit IgG (H+L) purchased from Beyotime (Shanghai, China) Pierce ECL Western Blotting Substrate was purchased from Thermo Scientific (Waltham, MA, USA).

### One-Step Growth Curve

IPEC-J2 cells were infected with 1 MOI (multiplicity of infection) of PEDV or TGEV at 12, 24, 36, 48, and 60 hpi. The supernatant was collected, followed by the 50% tissue culture infectious dose (TCID_50_) evaluation with the Reed Muench method as follows.

Briefly, Vero (for PEDV) or ST (for TGEV) cells were cultured in 96-well plates at a density of 1 × 10^5^ cells/well for 12 h, followed by washing with PBS three times. The collected supernatant was 10-fold diluted (10^-1^ to 10^-10^) with cell maintenance solution containing trypsin (final concentration of 10 μg/ml). Thereafter, cells were inoculated with the diluted virus at 37°C, 5% CO_2_ for 12, 24, 36, 48, and 60 hpi, and the cytopathic effect (CPE) was observed daily using an inverted microscope. TCID_50_ of each virus was calculated as described by Reed and Muench (26).

### Virus Infection

IPEC-J2 cells (2 × 10^5^/ml) were plated in 6-well plates, incubated overnight to reach 70%–80% confluency. Then, cells were inoculated with PEDV (MOI = 1), TGEV (MOI = 1), or PEDV+TGEV (MOI = 1 for each virus) supplemented with 10 μg/ml trypsin and cultured at 37, 5% CO_2_ for 12, 24, and 48 h. Cells were collected for lysis and extraction of RNA.

A549, BHK21, and DF-1 cells were transfected with pCAGGS-sIFITM3-Flag using Lipofectamine 3000 reagent (Thermo Fisher Scientific, USA) according to the manufacturer’s instruction. 24 h post-transfection, the expression of IFITM3 was examined by Western blot with anti-FLAG antibody. Then the cells were infected with rVSV-GFP at 0.1 MOI and the replication of rVSV-GFP was analyzed by examining *via* fluorescence microscope and flow cytometry at 24 hpi.

### RNA Extraction

Total RNA was extracted from virus-infected cells or mock cells using TRIzol Reagent according to the manufacturer’s instruction. The total RNA was dissolved in 50 μl of RNase-free ddH_2_O and stored at -20°C.

### Real-Time Quantitative PCR

Reverse transcription was performed using M-MLV Reverse Transcriptase RNase according to the manufacturer’s instruction. Thereafter, SYBR Green quantitative real-time PCR was performed using the ABI7500 Real-Time PCR Detection System and the GoTaq^®^ kit. The real-time PCR primers are listed in **Supplemental Table 1**. For each sample, the GAPDH gene was amplified and used as an internal control. The relative transcript levels of target genes were equal to the 2^(-ΔΔCt)^ threshold method and were shown as fold changes relative to the respective untreated control samples.

### RNA-Seq Analysis

To prepare the cDNA library, total RNA was treated with RNase-free DNase I. Then, mRNA was purified using magnetic oligo (dT) beads and evaluated using the Agilent 2100 bioanalyzer (Agilent Technologies, Santa Clara, CA, USA) for RNA integrity. mRNAs with RNA integrity numbers (RINs) > 8 were subjected to subsequent analysis. The purified mRNA was used to construct libraries using TruSeq PE Cluster Kit v3-cBot-HS (Illumina, San Diego, CA, USA) according to the manufacturer’s instructions. Then, these libraries were sequenced on an Illumina Novaseq platform (Illumina, USA).

### GO and KEGG Enrichment Analysis

Gene Ontology (GO) enrichment and Kyoto The Encyclopedia of Genes and Genomes (KEGG) pathway analysis of DEGs were conducted according to the protocols described by Cao et al. and Xie et al. previously (27, 28). Briefly, GO functional enrichment was performed using the Blast2GO software; the enriched genes were further classified by the GO analysis, with a p-value < 0.05.

The KEGG pathway database was accessed using the KOBAS software *via* a hypergeometric distribution test with the Phyper function in the R software package. Significantly enriched unigenes were selected based on a corrected p-value < 0.05. The distribution of DEGs within each GO/pathway category was determined by mapping all DEGs to terms in the GO and KEGG databases.

### STRING Pathway Analysis

GO and KEGG pathway enrichment analyses were analyzed by STRING (https://string-db.org/). The protein list was submitted for multiple protein searches. GO terms and KEGG pathway results were exported in the STRING analysis module. Terms and pathways with p <0.05 were significantly enriched. Appropriate terms and pathways were manually selected for visualization.

### Overexpression or Knockdown of IFITM3

IPEC-J2 cells were seeded in 6-well plates at a density of 2 × 10^5^/ml overnight to reach 70%–80% confluency. Then, cells were transfected with 4 μg of pLV-sIFITM3-Flag using Lipofectamine 3000 or 50 μM siRNAs targeting sIFITM3 (si-ssc-IFITM3_001, si-ssc-IFITM3_002, si-ssc-IFITM3_003) (**Supplemental Table 1**) using Lipofectamine RNAiMAX Reagent for 48 h.

For Vero cells, cells were transfected with 4 μg of pLV-sIFITM3-Flag using Lipofectamine 3000 or 50 μM siRNAs targeting monkey IFITM3 (si-csa-IFITM3_001, si-csa-IFITM3_002, si-csa-IFITM3_003) (**Supplemental Table 1**).

### Western Blot

Cells were harvested in IP lysis buffer containing the proteinase inhibitor cocktail (Sigma), frozen-thawed, and centrifuged to remove insoluble components. The total protein concentration was determined using a BCA protein assay kit (Beyotime Biotechnology, China). Protein samples were separated with 12% sodium dodecyl sulfate-polyacrylamide gel electrophoresis (SDS-PAGE) and transferred onto PVDF membranes, followed by blocking in 5% non-fat milk. Then, the membranes were incubated with antibodies in TBST containing 5% non-fat milk overnight at 4°C or 1 h at room temperature. After washing three times with TBST, the membranes were incubated with HRP-labeled goat anti-rabbit (or anti-mouse) IgG(H+L) IgG secondary antibodies (Beyotime Biotechnology) at room temperature for 30 min. Followed by washing, the membrane was visualized using Pierce ECL Western Blotting Substrate (Thermo Scientific).

### Flow Cytometry (FCM) Analysis

After trypsin incubation, the transfected cells were collected and washed with PBS twice. The cells were centrifuged at 3,000 rmp, at 4°C for 5 min, and subsequently resuspended in 5% PBS buffer at 4°C for 30 min. After centrifugation, the cells were incubated with the mouse anti-PEDV N mAb (FITC) in PBS buffer at 4°C for 30 min. After washing with PBS 3 times, the cells were resuspended in 200 ml PBS buffer at least 2.0 × 10^4^ cells per sample. Fluorescent intensity was determined and analyzed on CytoFLEX (Beckman, Brea, CA, USA).

### Crystal Violet Staining Assay

The Vero cells were washed with distilled water 3 times, fixed with 4% paraformaldehyde at room temperature for 20 min. After washing with distilled water 3 times, cells were stained with 0.1% crystal violet at room temperature for 15 min. The stained cells were washed with distilled water and air-dried for taking macrographic images.

### Statistical Analysis

The Student’s t-test was used for all experiment analyses. Data are presented as the mean ± standard deviation (SD) of 3 times experiments. p-values < 0.05 were considered statistical significance.

## Results

### Phylogenetic Analysis and Proliferation Kinetics of PEDV and TGEV in IPEC-J2 Cells

**Figure 1:**
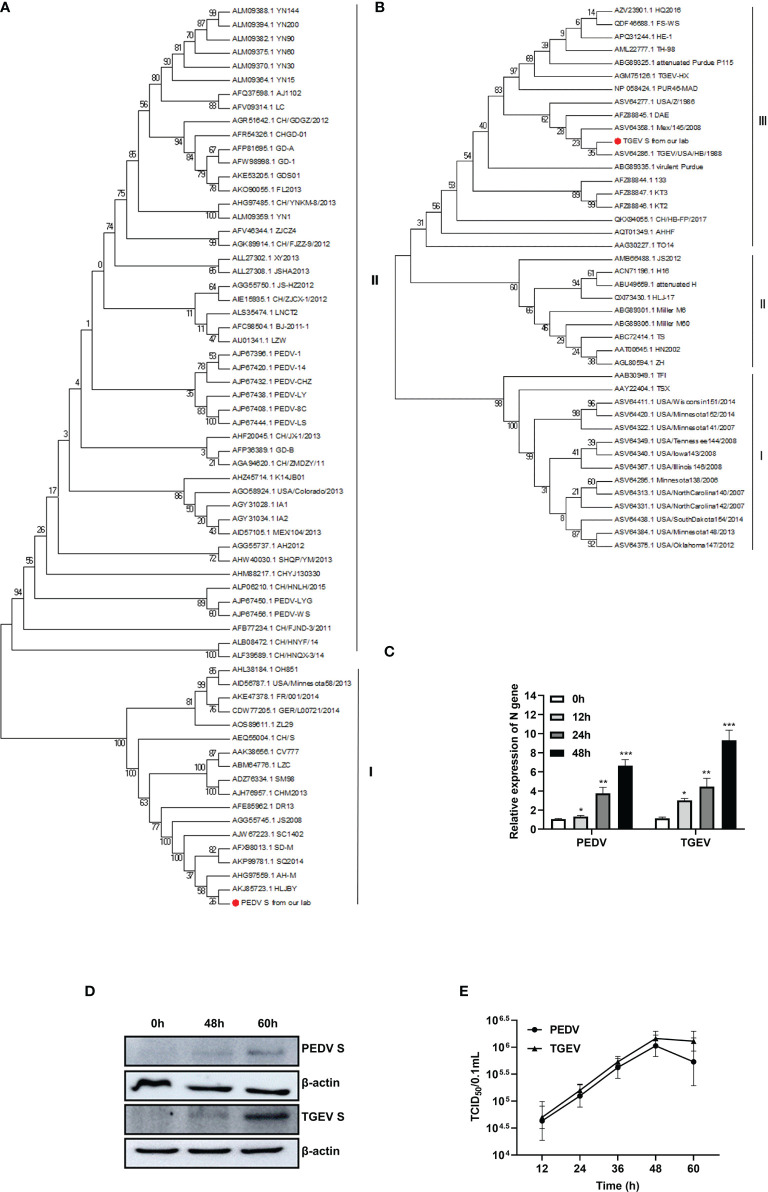
Phylogenetic analysis and proliferation kinetics of PEDV and TGEV in IPEC-J2 cells. **(A)** Phylogenetic trees of PEDV based on the S gene. **(B)** Phylogenetic trees of TGEV based on the S gene. **(C)** qPCR analysis of PEDV and TGEV infection in IPEC-J2 cells. IPEC-J2 cells were infected (MOI = 1) of PEDV or TGEV. The cells were collected at 0, 12, 24, 36, and 48 hpi respectively, followed by RT-PCR analysis. *, p-value <0.05; **, p-value <0.01; ***, p-value <0.001. **(D)** Western blot analysis of PEDV and TGEV replication in IPEC-J2 cells at 48 and 60 hpi. Unprocessed original images is found in **Supplementary Figure S1**. **(E)** One-step growth curve of PEDV and TGEV in Vero or ST cells, respectively. Viruses were collected from IPEC-J2 cells, followed by TCID_50_ evaluation.

Phylogenetic analysis of the PEDV and TGEV strains from our lab was constructed based on the S gene and is depicted in **Figure 1**. The PEDV strain was clustered with the PEDV classic strains (G1 cluster) (**Figure 1A**), whereas the TGEV strain was clustered into group III (**Figure 1B**).

To determine the infectivity and kinetics of the PEDV and TGEV propagation in the IPEC-J2 cells, levels of viral genes and viral titers were monitored after the virus infection. As shown in **Figures 1C, D**, both viruses were gradually increased in IPEC-J2 cells (**Figure 1C**). The results of Western blot showed that spike proteins of PEDV and TGEV were detected at 48 and 60 hpi (**Figure 1D**). Moreover, the titer of two strains was evaluated in Vero (for PEDV) or ST (for TGEV) cells, respectively. These results demonstrated that the titer of two strains at 48 hpi exceeded 10^6^/0.1 ml of TCID_50_ (**Figure 1E**). These results indicate that both viruses can effectively replicate in IPEC-J2 cells.

### Transcriptional Profile in IPEC-J2 Cells Induced by PEDV, TGEV, and PEDV+TGEV

**Figure 2:**
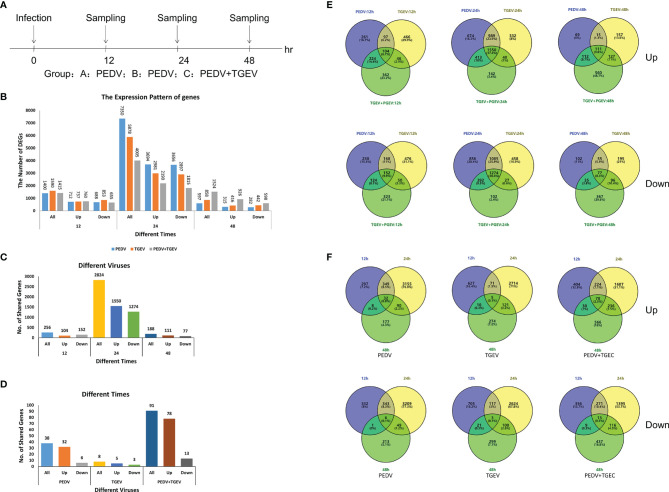
The differentially expressed genes in all transcriptome groups. **(A)** Schematic diagram of sampling. **(B)** The number of DEGs at different times. **(C, E)** DEGs of different groups at different times. **(D, F)** DEGs of the coinfected group at different times.

Cells were infected with PEDV, TGEV, and PEDV+TGEV, followed by sampling at 12, 24, and 48 hpi for whole genomic transcriptomics analysis (NCBI Accession No.: PRJNA796631, **Figure 2A**).

In total, 24,617 different genes were annotated from the transcriptome data, including 12,731 upregulated genes and 11,886 downregulated genes (**Supplemental Table 2**). As shown in **Figure 2B** and **Supplemental Table 2**, the total differential genes of PEDV, TGEV, and PEDV+TGEV were 1,400, 1,590, and 1,415 in three groups at 12 hpi, respectively, which was more than previously reported by Hu et al. (29). This shows that the amount of data in this study is more abundant than previous reports. At 24 hpi, the total differential genes of PEDV, TGEV, and PEDV+TGEV were 7,350, 5,878, and 4,005, respectively, while at 48 hpi, the total differential genes of PEDV, TGEV, and PEDV+TGEV were 597, 858, and 1,524, respectively. Notably, no matter the single infection or co-infection, the numbers of up- and downregulated DEGs in 24 hpi were more than that in 12 and 48 hpi, demonstrating that the interaction between virus and cell reached the maximized at 24 hpi.

Furthermore, the DEGs of the different viruses at different times were different. The shared DEGs were the most at 24 hpi, which was consistent with the above results (**Figure 2C**). Moreover, at the same time point, the shared DEGs of PEDV+TGEV-coinfected cells, including 78 upregulated DEGs and 12 downregulated DEGs, were more than those of the single infected groups (**Figure 2D**), indicating that coinfection of PEDV+TGEV may stimulate more DEGs.

Moreover, unique and shared DEGs in the coinfected group were analyzed *via* a Venn diagram. Both up- (1550) and downregulated (1,274) shared DEGs at 24 hpi were obviously increased in the PEDV+TGEV-coinfected group than that of the coinfected groups at 12 and 48 hpi (**Figure 2E** and **Supplemental Table 2**). Interestingly, the shared DEGs of the same infection groups at different times are less than the former (**Figure 2F** and **Supplemental Table 2**). These results suggest that the time point with the most DEGs was 24 hpi. Therefore, we focused on the DEGs in the coinfected cells at 24 hpi and analyzed the biological processes and molecular functions of upregulated genes and downregulated genes in the following studies.

### GO and KEGG Pathway Enrichment Analysis of the Shared DEGs

**Figure 3:**
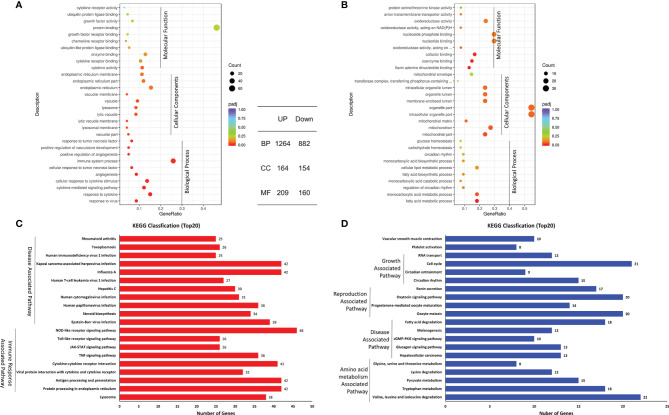
Top 20 pathways of the shared DEGs. **(A, B)** GO annotation of up- **(A)** and downregulated **(B)** DEGs. **(C, D)** KEGG analysis of up- **(C)** and downregulated **(D)** DEGs.

To analyze the function of the shared DEGs in the coinfected cells at 24 hpi, GO enrichments were performed and possible biological interactions of DEGs were examined. The results of GO analysis showed that 2,833 DEGs were identified (**Figure 3A**), including 1,637 upregulated DEGs and 1,196 downregulated DEGs. Furthermore, 2,146 of 2,833 DEGs belonged to the biological process (BP), with 1,264 upregulated and 882 downregulated. 318 of 2,833 DEGs were cellular components (CC), containing 164 upregulated and 154 downregulated. 369 of 2,833 DEGs were molecular function (MF), including 209 upregulated and 160 downregulated. The most annotated GO terms were protein binding (MF), immune system process (BP), organelle part (CC), intracellular organelle part (CC), etc. (**Figures 3A, B** **)**. These results indicate that the biological process and molecular function of the upregulated and downregulated DEGs were different in the coinfected cells at 24 hpi.

Moreover, KEGG classification showed that the upregulated DEGs included Disease-Associated Pathway and Immune Response Associated Pathway, while the downregulated DEGs were annotated to Growth-Associated Pathway, Disease-Associated Pathway, Reproduction-Associated Pathway, and Amino acid metabolism-Associated Pathway (**Figures 3C, D** **)**. The enriched pathways of upregulated DEGs were inconsistent with those of the downregulated DEGs.

### Evaluation of the Interferon-Stimulated Genes

Interferon-stimulated genes (ISGs) are molecules regulated by interferon, which has important influences on the host’s natural immunity and virus infection. Therefore, we further analyzed the ISGs of the shared genes. Among the upregulated DEGs, 90 ISGs were identified in this study, which were associated with PEDV or TGEV infection (**Supplemental Table 3**). Based on the biological function, the identified ISGs can be classified into several groups, including antiviral, antigen presentation, AMP sensing+IFN pathway, miscellaneous, cell signaling and apoptosis, and Ubiquitin-related groups (**Supplemental Table 3**).

**Figure 4:**
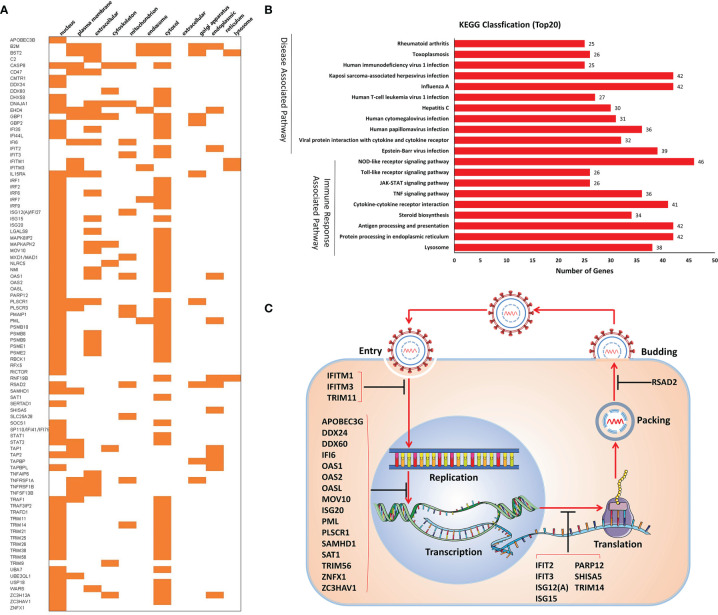
Analysis of 90 ISGs. **(A)** Subcellular localization. **(B)** KEGG classification. **(C)** Antiviral activities of ISGs.

Furthermore, the subcellular location of the ISGs demonstrated that most of the ISGs were in the nucleus (68 ISGs) and cytosol (58 ISGs) (**Figure 4A**). Enrichment analysis showed that the ISGs were involved in virus infection and immune response-related pathways, including NOD-like/RIG-I/Toll-like receptor signaling pathways, JAK-STAT signaling pathway, antigen processing and presentation, and pathways induced by other viruses’ infection (**Figure 4B**). Especially, 27 ISGs play antiviral roles in these ISGs (**Table 1**). As shown in **Figure 4C**, these ISGs inhibit or delay the process of virus proliferation in different infection stages, such as entry, replication, transcription and translation, packing, and budding. Notably, most of the ISGs mainly target the replication–transcription complex/system. These results indicate that these ISGs are suitable candidate targets for antiviral research.

**Table 1:** Antiviral interferon-induced genes in this study.

Gene symbol	PEDV infection		TGEV infection		PEDV+TGEV infection		Gene description	Ref DOI
	log2 fold change	q value	log2 fold change	q value	log2 fold change	q value		
APOBEC3B	0.853622611	3.47E-09	0.716693607	0.002364504	0.953401477	1.82169E-06	Apolipoprotein B mRNA editing enzyme catalytic subunit 3B	10.1038/nature00939
DDX24	0.227246637	0.039698472	0.295532008	0.04570764	0.304417963	0.020049853	DEAD-box helicase 24	10.1016/j.virol.2008.01.025
DDX60	1.110014124	0.048911194	0.998530685	0.032603554	0.955513667	0.013291317	DExD/H-box helicase 60	10.1038/nature09907
IFI6	1.479421789	0.000611615	1.864487534	5.95363E-07	1.377998512	3.09053E-05	Interferon alpha inducible protein 6	10.1038/s41564-018-0244-1
IFIT2	2.292753267	0.005583963	2.170059221	0.010245215	2.269357635	0.007031308	Interferon-induced protein with tetratricopeptide repeats 2	10.1371/journal.pbio.2004086
IFIT3	1.897685589	0.008779897	1.658142819	0.001373656	1.911377054	0.011643211	Interferon-induced protein with tetratricopeptide repeats 3	10.1371/journal.pbio.2004086
IFITM1	2.188304133	7.74E-08	2.519998152	1.35833E-10	1.987652389	1.70188E-10	Interferon-induced transmembrane protein 1	10.1016/j.cell.2009.12.017
IFITM3	1.132823871	4.43E-10	1.434383357	3.99482E-06	1.113465091	9.84344E-07	Interferon-induced transmembrane protein 3	10.3389/fimmu.2018.00228
ISG12(A)	1.822958691	0.00000265	2.186524528	7.21423E-08	1.614782083	3.25923E-07	Putative ISG12(a) protein	10.1128/JVI.00352-16
ISG15	2.302352998	0.00000341	2.663277381	6.77695E-09	2.05115159	3.77527E-08	ISG15 ubiquitin-like modifier	10.1371/journal.pbio.2004086
ISG20	1.742182074	0.000108971	1.948342895	1.72743E-06	1.502146139	3.99749E-06	Interferon stimulated exonuclease gene 20	10.1371/journal.pbio.2004086
MOV10	1.493423741	2.6E-18	1.639326559	1.46511E-08	1.205502595	1.4368E-07	Mov10 RISC complex RNA helicase	10.1371/journal.pbio.2004086
OAS1	1.332989798	2.97E-09	1.421244578	1.39396E-05	1.215590342	1.36733E-05	2′-5′-Oligoadenylate synthetase 1	10.1371/journal.pbio.2004086
OAS2	1.206408548	4.93E-08	1.117235101	0.000354723	1.127790413	5.62295E-05	2′-5′-Oligoadenylate synthetase 2	10.3390/v12040418
OASL	2.319409818	0.000176627	2.883551358	1.62918E-09	2.127805209	0.001844082	2′-5′-pligoadenylate synthetase like	10.1016/j.immuni.2018.12.013
PARP12	1.21350878	0.00365218	1.225430781	0.000345214	1.012772207	0.001336873	Poly(ADP-ribose) polymerase family member 12	10.1371/journal.pbio.2004086
PLSCR1	0.843484336	0.000034	0.81086527	0.004374285	0.825097418	0.001428997	Phospholipid scramblase 1	10.1128/JVI.78.17.8983-8993.2004.
PML	1.110555091	4.43E-08	1.189068424	7.19109E-05	1.117956227	9.76855E-06	Promyelocytic leukemia	10.1371/journal.pbio.2004086
RSAD2	1.739470018	0.025127966	1.263835725	0.026307427	1.743586308	0.032480782	Radical S-adenosyl methionine domain-containing 2	10.1016/j.virusres.2019.01.014
SAMHD1	1.57993947	0.0000295	1.561018618	1.70923E-05	1.419359396	1.79687E-05	SAM and HD domain-containing deoxynucleoside triphosphate triphosphohydrolase 1	10.1016/j.tim.2015.08.002
SAT1	1.234166325	0.000000407	1.816910382	4.24858E-08	1.202642104	0.000799724	Spermidine/spermine N1-acetyltransferase 1	10.1371/journal.pbio.2004086
SHISA5	1.013779953	3.45E-10	1.139773973	9.82032E-06	0.854640948	2.51898E-05	Shisa family member 5	10.1038/ncomms10631
TRIM11	0.79732917	0.000000202	0.859346252	0.000532122	0.642531515	0.000439271	Tripartite motif containing 11	10.1371/journal.ppat.0040016
TRIM14	0.929915084	0.00000209	0.638263983	0.023485833	1.033985153	5.19874E-05	Tripartite motif containing 14	10.3389/fmicb.2019.00344
TRIM56	1.109190465	7.78E-08	0.868601165	0.017945664	0.908904097	0.000233247	Tripartite motif containing 56	10.1371/journal.pntd.0007537
ZC3HAV1	1.260902829	3.74E-09	0.78703522	0.021422393	1.01385866	9.58972E-05	Zinc finger CCCH-type antiviral protein 1	10.1371/journal.pbio.2004086
ZNFX1	1.244001392	0.016690307	0.937197617	0.021673342	1.272066064	0.000428093	Zinc finger NFX1-type containing 1	10.1038/s41556-019-0416-0

**Figure 5:**
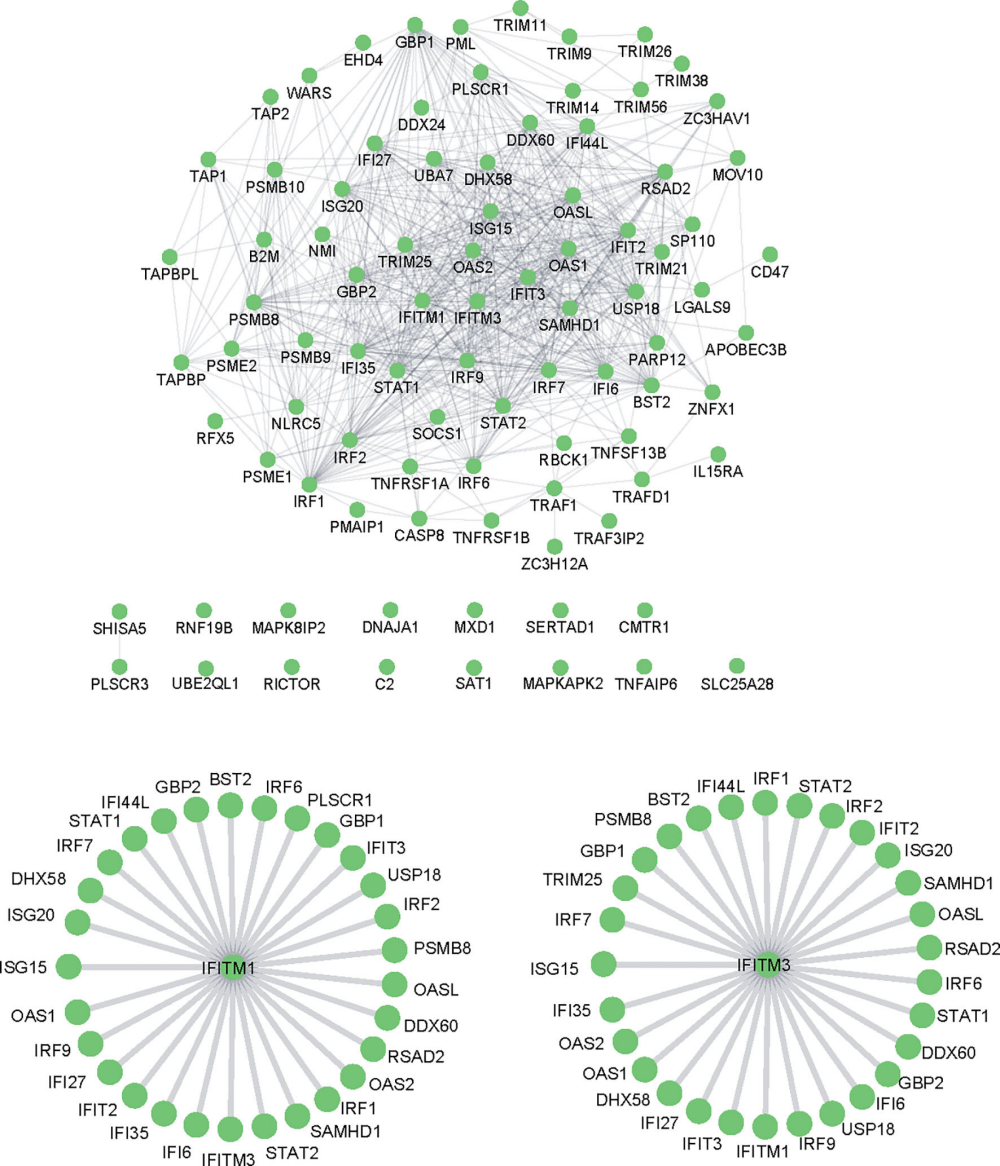
Protein–protein interaction networks.

Moreover, STRING analysis was used to assess the potential interaction network of the ISGs related to response to the virus. As shown in **Figure 5A**, most ISGs interacted with other proteins to form a complex protein–protein interaction network. It is worth noting that interferon-induced transmembrane proteins (IFITMs), especially IFITM1 and IFITM3, were also included in the identified ISGs, which have been widely studied for their antiviral mechanism in the last decade. Among the ISGs, IFITM1 (**Figure 5B**) and IFITM3 (**Figure 5C**) exerted antiviral activities by interacting with various ISGs, which suggests that IFITM1 and IFITM3 are critical in resisting virus immune responses. Therefore, we focused on IFITMs to clarify whether these molecules are involved in the antiviral activities against PEDV and TGEV infection.

### Confirmation of the Identified ISGs by Real-Time qPCR

**Figure 6:**
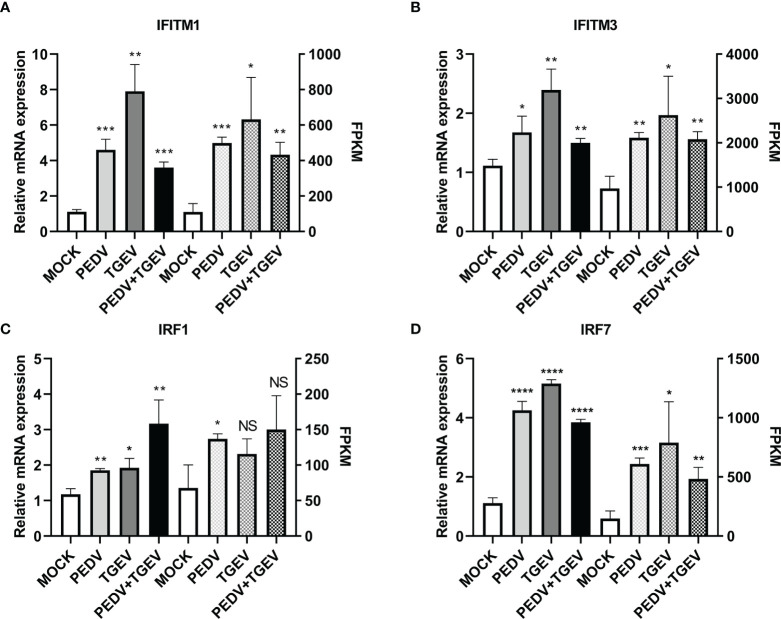
Differentially expressed key genes. IPEC-J2 cells were infected with 1 MOI of PEDV, TGEV, and PEDV+TGEV for 24 hpi. The expression levels of IFITM1 **(A)**, IFITM3 **(B)**, IRF1 **(C)**, and IRF7 **(D)** were compared at 24 hpi with RT-PCR. GAPDH was used as the internal control. *, p-value <0.05; **, p-value <0.01; ***, p-value <0.001; ****, p-value <0.0001. NS, no significant.

To further confirm the above results, levels of ISGs with antiviral activities, such as IFITM and IRF genes, were evaluated using real-time PCR. As shown in **Figure 6**, IFITM1, IFITM3, IRF1, and IRF7 genes were significantly upregulated at 24 hpi compared with that of the mock-infected group, which were consistent with the RNA-seq data.

### Knocking Down IFITMs Enhanced Virus Infection, While Overexpression of IFITMs Inhibited Virus Infection

**Figure 7:**
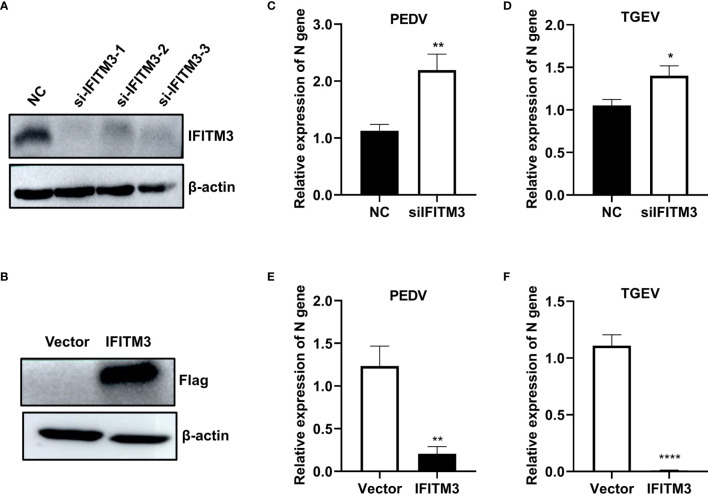
Evaluation of PEDV and TGEV in IFITM3-overexpressed and knocked-down cells. Porcine IPEC-J2 cells were transfected with si-ssc-IFITM3s or pLV-sIFITM3-Flag for 48 h, followed by infection with PEDV or TEGV (MOI = 1). The expressions levels of sIFITM3 were evaluated by Western blot at 48 h post-transfection **(A, B)**, and the levels of viral genes were quantified at 24 h postinfection using real-time PCR **(C–F)**. **(A, C, D)** IPEC-J2 cells transfected with siIFITM3s. **(B, E, F)** IPEC-J2 cells transfected with pLV-sIFITM3-Flag. Unprocessed original images are found in **Supplementary Figure S2**. *, p-value <0.05; **, p-value <0.01; ****, p-value <0.0001.

To evaluate the effect of IFITM3 on PEDV and TGEV infection, porcine IPEC-J2 cells were transfected with si-ssc-IFITM3s or pLV-sIFITM3-Flag for 48 h, followed by infection with PEDV or TEGV (MOI = 1). The results showed that the expressions levels of sIFITM3 (sIFITM3) were significantly decreased in the si-ssc-IFITM3s-transfected cells and increased in the pLV-sIFITM3-Flag-transfected cells compared to that of the control groups (**Figures 7A, B** **)**. Expectedly, the expression levels of PEDV and TEGV genes were significantly increased in the si-ssc-IFITM3s-transfected cells (**Figures 7C, D** **)** and decreased in the pLV-sIFITM3-Flag-transfected cells compared to that of the control groups (**Figures 7E, F** **)**. These results indicate that sIFITM3 has antiviral activity against PEDV and TGEV infection.

**Figure 8:**
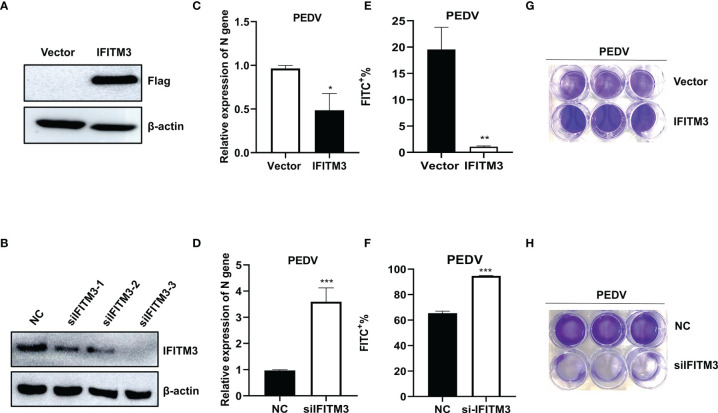
Proliferation of PEDV in IFITM3-overexpressed and knocked-down Vero cells. Vero cells were transfected with pLV-sIFITM3-Flag or si-csa-IFITM3s for 48 h, followed by infection with PEDV (MOI = 1) for 48 h. The proliferation of PEDV in IFITM3-overexpressed and knocked-down Vero cells was evaluated at 24 h postinfection using real-time PCR, flow cytometry assay, and crystal violet staining assay. **(A, B)** Western blot. **(C, D)** Real-time PCR. **(E, F)** flow cytometry assay. **(G, H)** Crystal violet assay. Unprocessed original images are found in **Supplementary Figure S3**. *, p-value <0.05; **, p-value <0.01; ***, p-value <0.001.

To further confirm the above results, Vero cells, a heterogeneous cell line, were transfected with pLV-sIFITM3-Flag or si-csa-IFITM3s for 48 h, followed by infection with PEDV (MOI = 1) for 48 h. As shown in **Figure 8**, the expression levels of IFITM3 were increased in the pLV-sIFITM3-Flag-transfected cells and significantly decreased in the si-csa-IFITM3s-transfected cells compared to that of the control groups (**Figures 8A, B** **)**. Meanwhile, the expression levels of PEDV genes were significantly decreased in the pLV-sIFITM3-Flag-transfected cells and increased in the si-csa-IFITM3s-transfected cells compared to that of the control groups (**Figures 8C, D** **)**. Furthermore, the proliferation of PEDV in IFITM3-overexpressed and knocked-down Vero cells was evaluated using flow cytometry assay and crystal violet staining assay. The results showed consistency with real-time PCR results (**Figures 8E, F** **)**. The cell viability was significantly increased in the sIFITM3-overexpressed and decreased in the knocked-down Vero cells compared with the control groups, respectively (**Figures 8G, H** **)**. These results further confirmed that knocking down IFITM3 enhanced virus infection, while overexpression of sIFITM3 inhibited virus infection.

**Figure 9:**
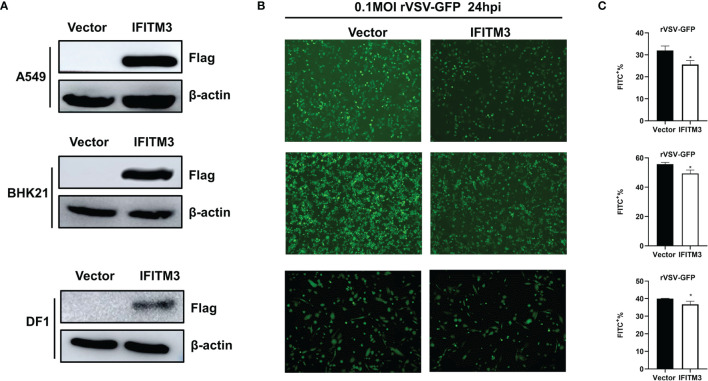
sIFITM3 antagonizes rVSV-GFP proliferation in different cells. A549, BHK21, and DF-1 cells were transfected with pCAGGS-sIFITM3-Flag using Lipofectamine 3000 reagent. 24 h post-transfection, the expression of IFITM3 was examined by Western blot **(A)** with anti-FLAG antibody. Then the cells were infected with rVSV-GFP at 0.1 MOI and the replication of rVSV-GFP was analyzed by examining *via* fluorescence microscopy **(B)** and flow cytometry **(C)** at 24 hpi. *, p-value <0.05.

Additionally, A549, BHK21, and DF-1 cells were transfected with pCAGGS-sIFITM3-Flag, followed by infection with rVSV-GFP. As shown in **Figure 9A**, the sIFITM3 can be efficiently expressed in these cells. The replication of rVSV-GFP was significantly inhibited in the sIFITM3-expressed cells (**Figures 9B, C** **)**. These results further suggest that the antiviral activity of sIFITM3 is broad-spectrum *in vitro*.

## Discussion

Porcine diarrhea-associated viruses including PEDV, TGEV, porcine deltacoronavirus (PDCoV), and porcine rotavirus (PoRV) are four common causative agents for viral diarrhea in pigs worldwide (1–3, 6, 7). During 2012–2020, PEDV and TGEV are the top two viruses reported from pig farms in China, responsible for porcine diarrhea and devastating economic losses to the swine industry. It was reported that both TGEV and PEDV infection can activate the JAK-STAT1 signaling pathway and ISGs (23, 30). Moreover, differential protein expressions were detected in cells infected with PEDV pandemic and classical strains, including antiviral pathways and proteins, such as RLRs, autophagy, MAPK pathways, and ISGs (30–33). Coinfection of TGEV and enterotoxigenic *Escherichia coli* K88 (ETEC) regulated host proteins, thus enhancing the persistence of pathogen infection, which was partly due to the inhibition of TGEV-induced inflammatory cytokines by ETEC (34). However, differentially expressed genes in cells coinfected with PEDV and TGEV have not been reported to date. In this study, we evaluated the comparative transcriptomics between PEDV and TGEV single and coinfection. The results showed that DEGs can be detected in the cells infected with PEDV, TGEV, and PEDV+TGEV at 12, 24, and 48 hpi, and the number of DEGs was the highest at 24 hpi (**Figure 2**). Furthermore, coinfection of PEDV+TGEV leads to more DEGs than that of the single infection, which mainly annotated to the GO terms of protein binding (BP), immune system process (MF), organelle part (CC), intracellular organelle part (CC), etc. (**Figure 3**). KEGG classification showed that the upregulated DEGs included Disease-Associated Pathway and Immune Response Associated Pathway (**Figure 3**). Therefore, we chose the shared DEGs associated with immune responses for further study.

Type I interferons, including IFN-α and IFN-β, are critical antiviral cytokines of host immune responses. However, the IFN responses induced by enteric coronaviruses in the intestinal epithelial cells are different from that of the other epithelial cells, which is partly due to the distinct characteristics of the intestinal epithelial mucosal surface and gut microflora (35). Meanwhile, type III interferon (IFN-λ) plays a vital role against infections of enteric coronaviruses (35–37). On the contrary, PEDV, especially nsp1, suppressed IFN-λ activities and interferon regulatory factor 1 (IRF1) signaling *via* inhibiting IRF1 nuclear translocation and reducing the number of peroxisomes, thus blocking the IRF1-mediated type III IFNs (37), suggesting that PEDV can escape the IFN-λ responses in intestinal epithelial cells. Furthermore, TGEV infection can stimulate endoplasmic reticulum (ER) stress and IFN-I production. However, TGEV can also evade the type I IFN antiviral response *via* IRE1α-mediated modulation of the miR-30a-5p/SOCS1/3 axis (38). Here, 90 ISGs were upregulated during PEDV or TGEV infection, which were subcellularly located in the nucleus and cytosol. Among the upregulated ISGs, 27 ISGs, including IRF1, IRF7, IFITM1, and IFITM3, play antiviral roles in different stages of virus proliferation, thus inhibiting or delaying the process of virus infection by interacting with other proteins or ISGs to form a complex protein–protein interaction network.

IFITMs are kinds of small-molecule transmembrane proteins induced by interferon, which are important restriction factors and play broad-spectrum antiviral activities (39–41). IFITMs mainly target viral-to-cellular membrane fusion to block the early stage of virus infection and/or trigger the production of novel virions with decreased infectivity (40). IFITMs can inhibit the feline foamy virus at the late step of viral replication (42). Meanwhile, IFITM1 also exerts antiviral activity by regulating host lipid metabolism (43). We previously found that IFITM3 inhibited vaccinia virus and thrombocytopenia syndrome virus (SFTSV) infection (44, 45). Another group reported that both IFITM1 and IFITM3 can be induced by IFN-α and IFN-λ in IPEC-J2 cells in a dose-dependent manner (36). However, functional heterogeneity was detected in mammalian IFITMs, and critical domains of IFITMs with antiviral activity were conserved among mammalian IFITMs (40). As reported, human and mouse IFITM1, IFITM2, and IFITM3 restricted SARS-CoV-2 infections with a distinct mechanism (46). The amphipathic helix and its amphipathic properties of IFITM3 were critical for virus restriction, but its mutation will be converted into an enhancer for SARS-CoV-2 infection and cell-to-cell fusion (46). Moreover, a recent report indicated that several viruses may escape IFN- and IFITM-mediated inhibition, especially cell-to-cell spread, leading to chronic and persistent infections and illness (47). Therefore, we further evaluated the antiviral activity of sIFITM3 in cells infected with PEDV and TGEV. As a result, sIFITM3 can significantly inhibit PEDV and TGEV infection in both porcine IPEC-J2 cells and monkey Vero cells. Also, sIFITM3 can significantly inhibit VSV-EGFP infection in different species cells, such as human A549 cells, mouse BHK21 cells, and avian DF-1 cells. These results further confirm that sIFITM3 has broad-spectrum antiviral activity. In addition, IFITMs are S-palmitoylated proteins in vertebrates that restrict a diverse range of viruses (48–50). S-palmitoylated IFITM3 in particular engages incoming virus particles, prevents their cytoplasmic entry, and accelerates their lysosomal clearance by host cells (48, 50). However, how S-palmitoylation modulates the structure and biophysical characteristics of IFITM3 to promote its antiviral activity remains unclear. The research on the mechanism of sIFITM3 inhibiting virus infection as well as the function of S-palmitoylated sIFITM3 in virus infection is still in progress.

## Conclusion

In this study, transcriptomes especially shared DEGs and ISGs are different in cells single or co-infected with PEDV and TGEV, suggesting that cells have different responses to virus infection. We firstly identified that sIFITM3 inhibits PEDV, TGEV, and VSV-EGFP, which suggests that sIFITM3 has broad-spectrum antiviral activity. Further studies are needed to elucidate the antiviral function and molecular mechanism of sIFITM3. Our research enriched the knowledge of cells against PEDV and TGEV infection and confirmed that IFITM3 is one of the important antiviral ISGs.

## Data Availability Statement

The datasets presented in this study can be found in online repositories. The names of the repository/repositories and accession number(s) can be found as follows: National Center for Biotechnology Information (NCBI) BioProject database under accession number PRJNA796631.

## Author Contributions

Conceptualization, NJ, CL, and LR. Methodology, LS and JC. Software, PH and YJ. Validation, WX and LL. Formal analysis, LS and SC. Data curation, LS and ZG. Writing—original draft preparation, LS, CL, and SC. Writing—review and editing, LR, CL, and NJ. Visualization, CL and SC. Supervision, LR, CL, and NJ. Project administration, CL. Funding acquisition, LR and NJ. All authors contributed to the article and approved the submitted version.

## Funding

This work was supported by the National Key Research and Development Program of China [No. 2021YFD1801103]; the National Natural Science Foundation of China [Nos. 31972719, 31772747]; the Jilin Province Science and Technology Development Project [No. 20200402043NC]; and the Jilin University Science and Technology Innovative Research Team [JLU-STIRT, 2017TD-05]. The funders had no role in study design, data collection and analysis, decision to publish, or preparation of the manuscript.

## Conflict of Interest

The authors declare that the research was conducted in the absence of any commercial or financial relationships that could be construed as a potential conflict of interest.

## Publisher’s Note

All claims expressed in this article are solely those of the authors and do not necessarily represent those of their affiliated organizations, or those of the publisher, the editors and the reviewers. Any product that may be evaluated in this article, or claim that may be made by its manufacturer, is not guaranteed or endorsed by the publisher.
