# Supplementary material for: Differential Transcriptomics Analysis of IPEC-J2 Cells Single or Coinfected With Porcine Epidemic Diarrhea Virus and Transmissible Gastroenteritis Virus
Source: Front Immunol. 2022 Mar 25;13:844657. doi: 10.3389/fimmu.2022.844657 (PMC8989846; doi:10.3389/fimmu.2022.844657)
Supplement: Supplementary file 5 [file DataSheet_5.docx]

**Differential transcriptomics analysis of IPEC-J2 cells single or co-infected with porcine epidemic diarrhea virus and transmissible gastroenteritis virus**

Lina Song^1,2^, Jing Chen^2^, Pengfei Hao^2^, Yuhang Jiang^2^, Wang Xu^2^, Letian Li^2^, Si Chen^3^, Zihan Gao^2^, Ningyi Jin^2🖂^, Linzhu Ren^3🖂^, and Chang Li^2 🖂^

^1^ College of Veterinary medicine, Key Lab for Zoonoses Research, Ministry of Education, Jilin University, Changchun 130062, China

^2^ Research Unit of Key Technologies for Prevention and Control of Virus Zoonoses, Chinese Academy of Medical Sciences, Changchun Institute of Veterinary Medicine, Chinese Academy of Agricultural Sciences, Changchun, China

^3^ College of Animal Sciences, Jilin University, Changchun 130062, China

**Running Title:** Differential transcriptomics of cells infected with PEDV and/or TGEV

^🖂^ Correspondence: lichang78@163.com (Li C); renlz@jlu.edu.cn (Ren L); [ningyik@126.com](mailto:ningyik@126.com) (Jin N)

**Fig S1: Original Western images used for preparing Figure 1.**

| PEDV-S | 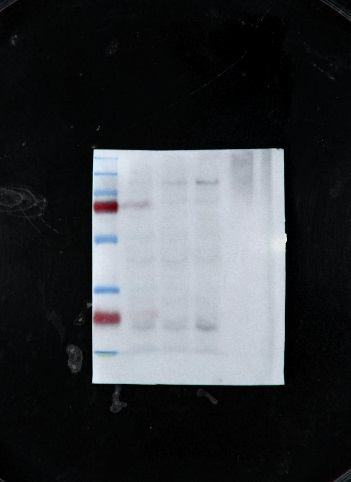 |
| --- | --- |
| ACTIN | 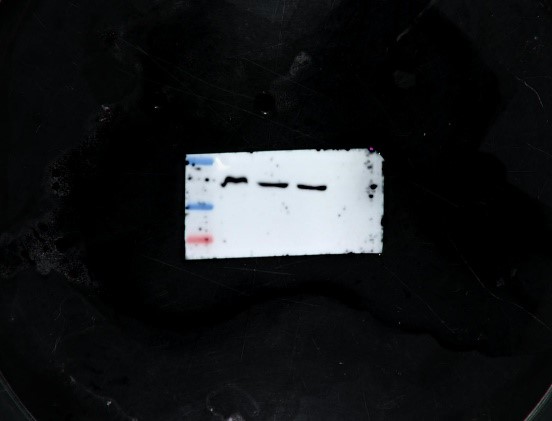 |
| TGEV-S | 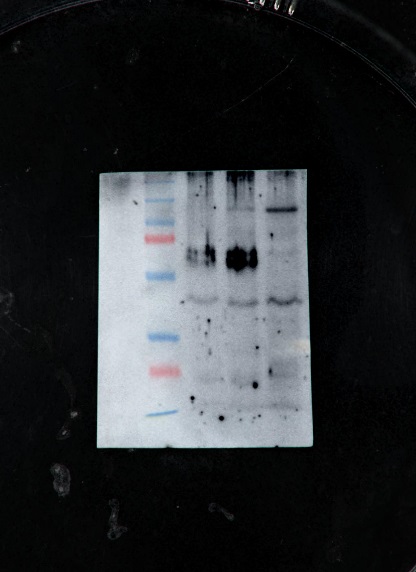 |
| ACTIN | 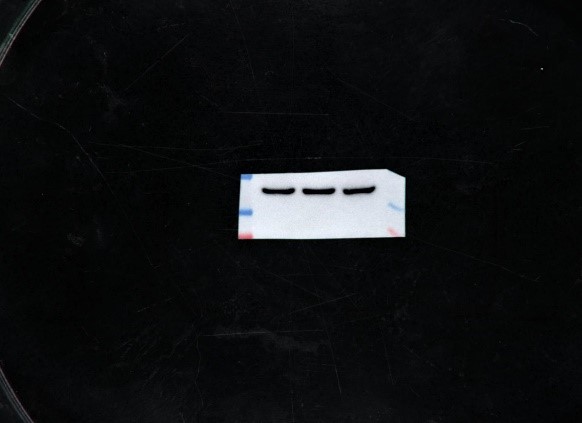 |

**Fig S2: Original Western images used for preparing Figure 7.**

| IFITM3 | 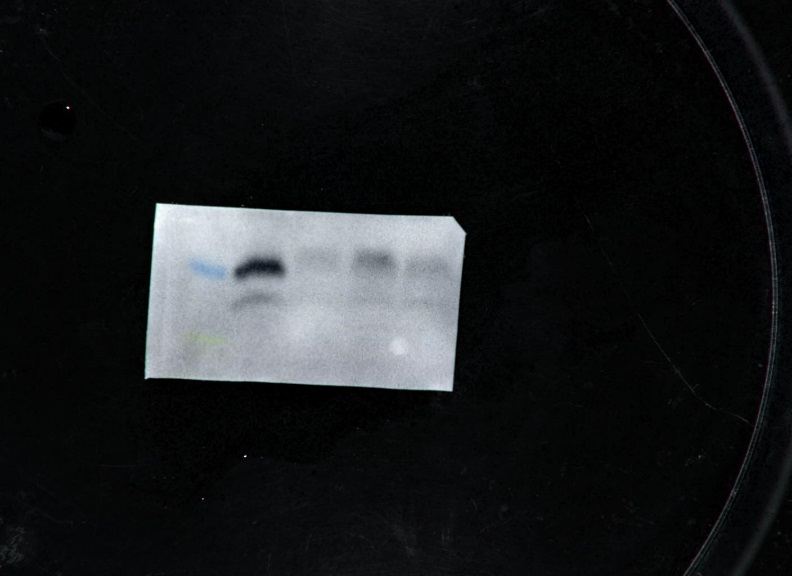 |
| --- | --- |
| ACTIN | 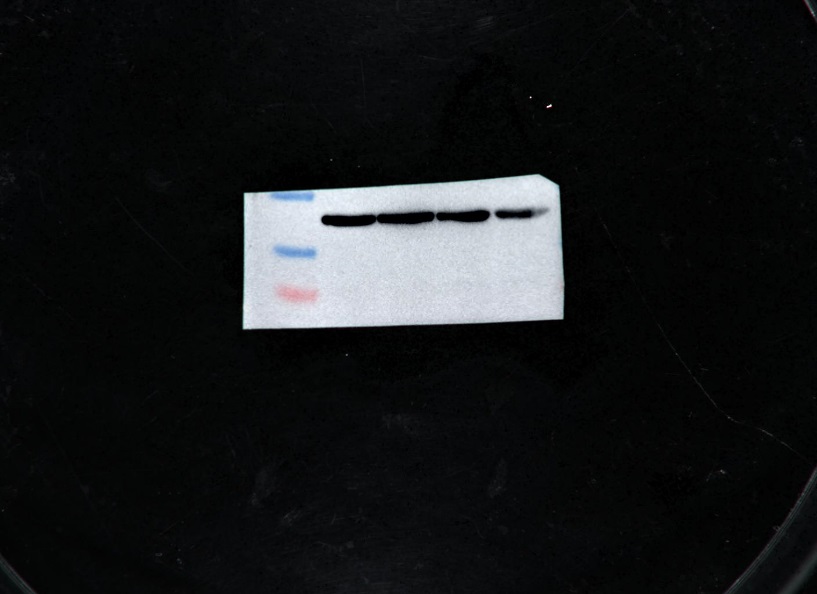 |
| Flag | 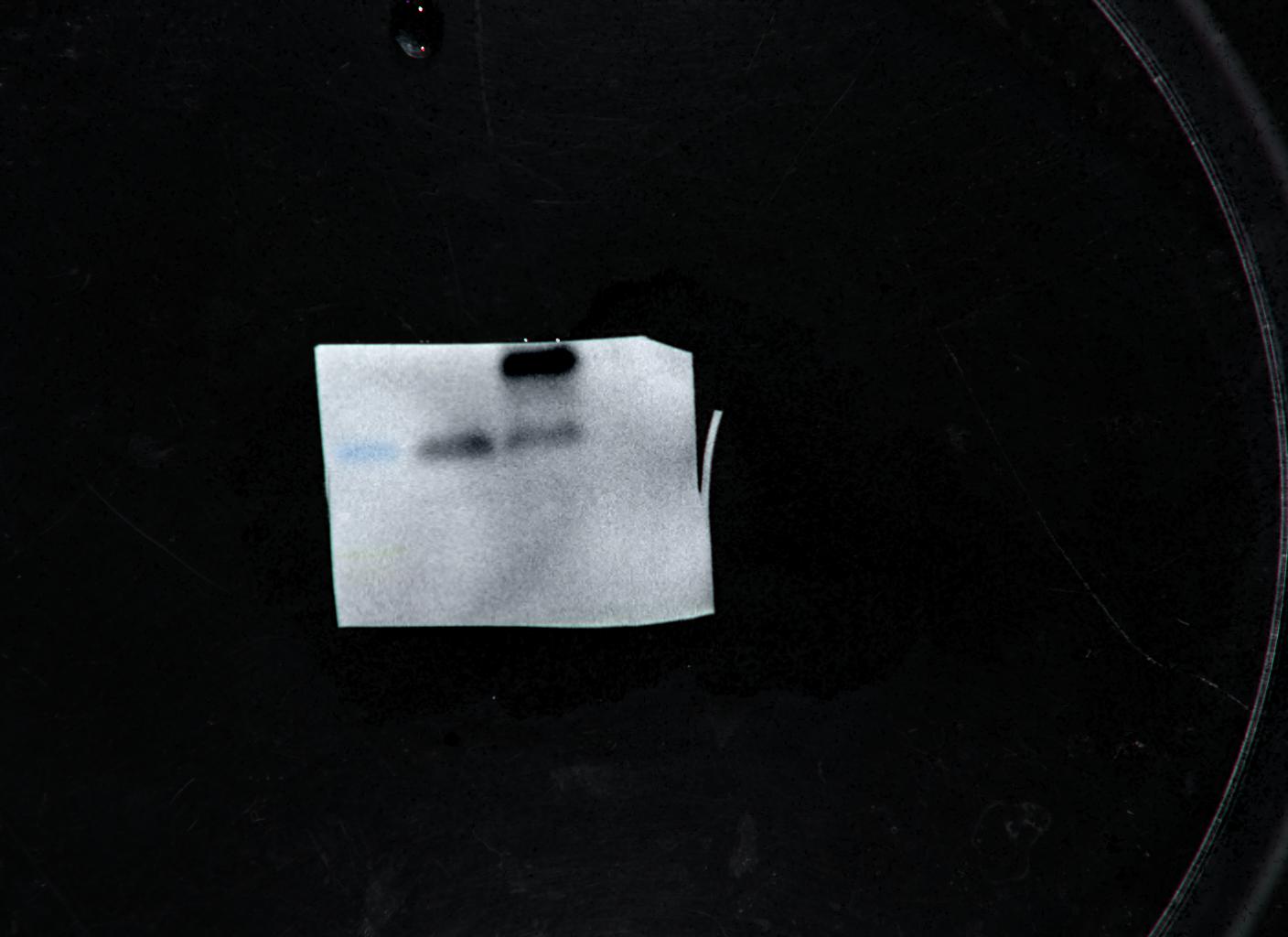 |
| ACTIN | 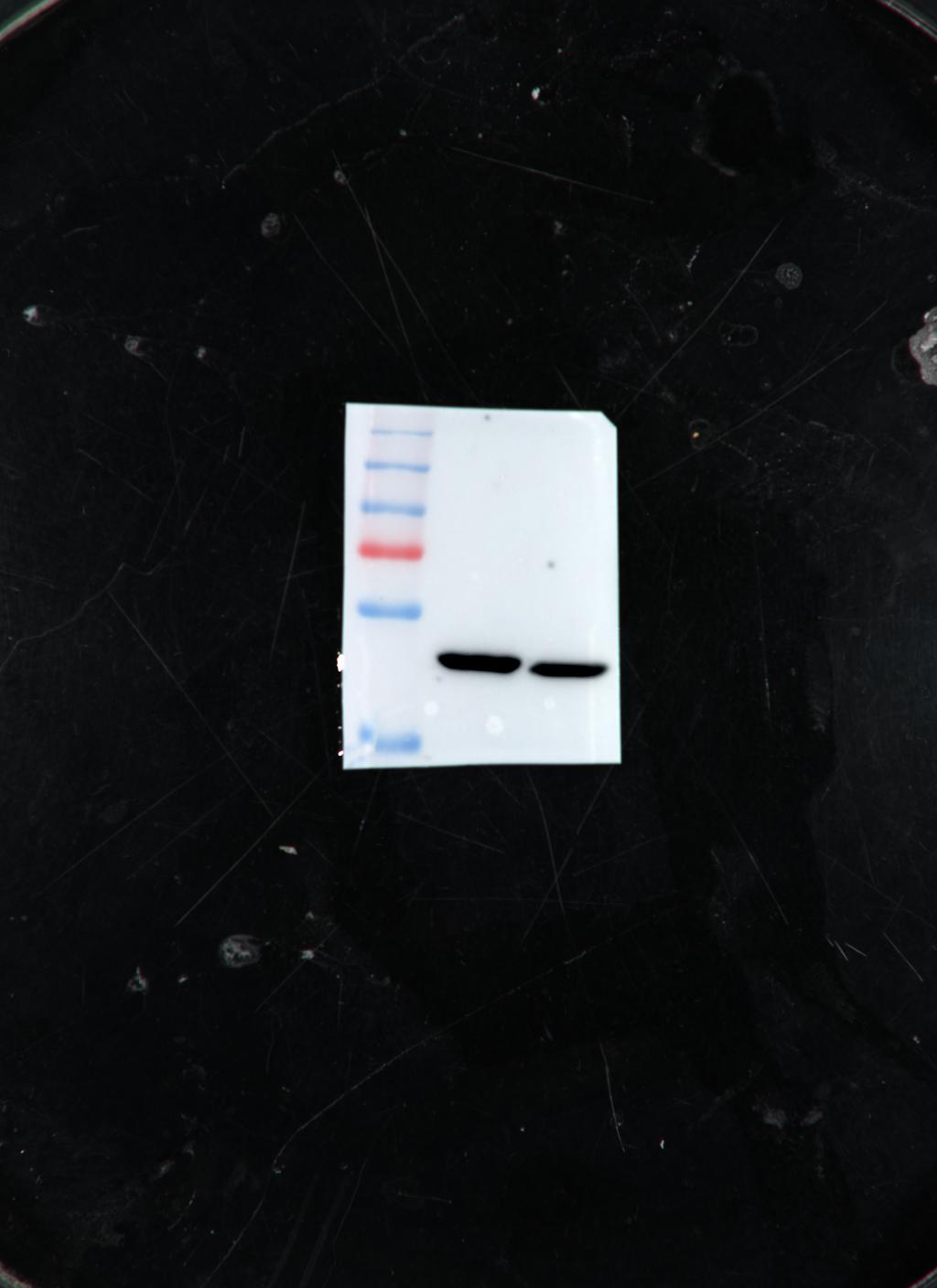 |

**Fig S3: Original Western images used for preparing Figure 8.**

| Flag | 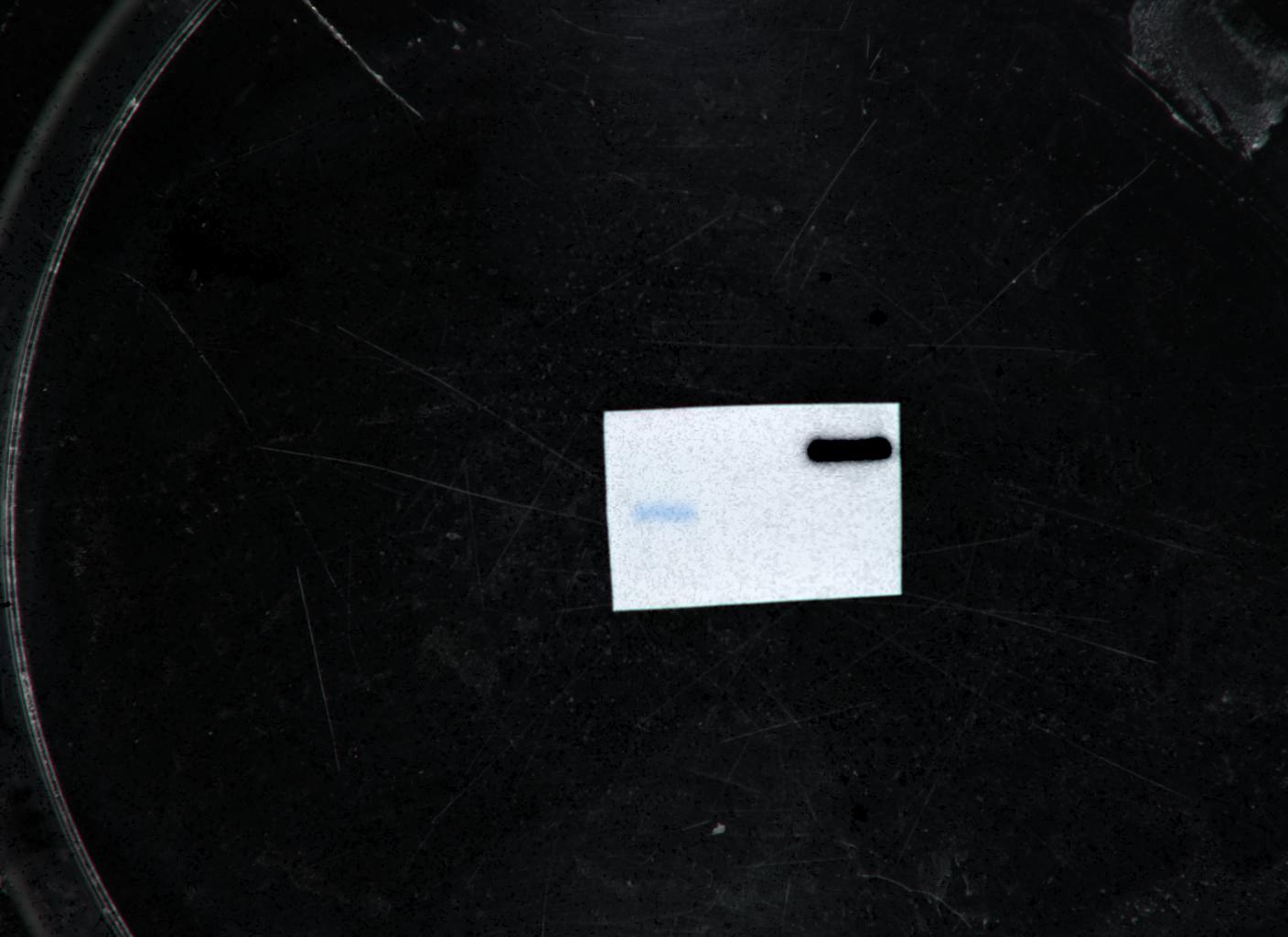 |
| --- | --- |
| ACTIN | 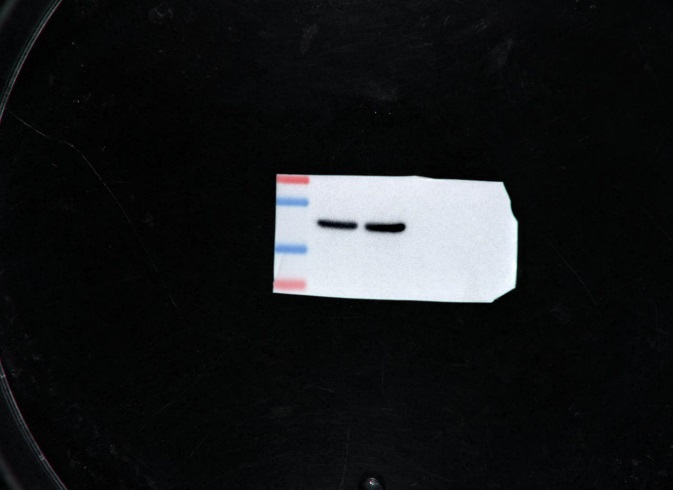 |
| IFITM3 | 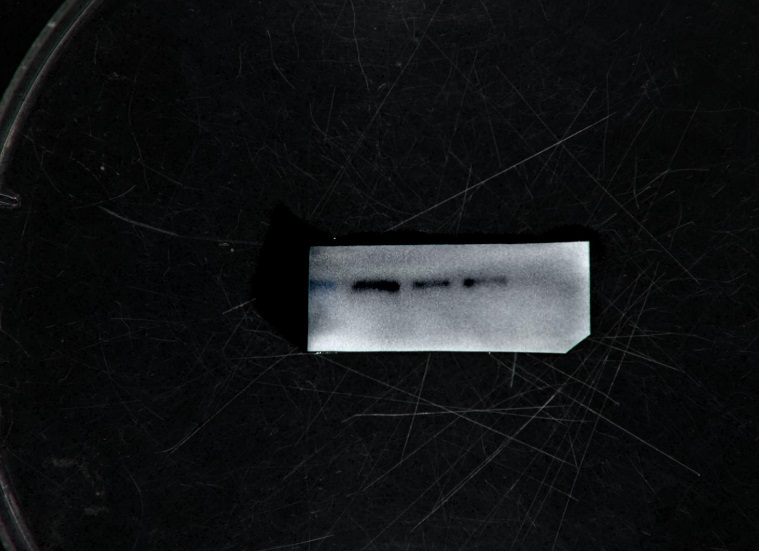 |
| ACTIN | 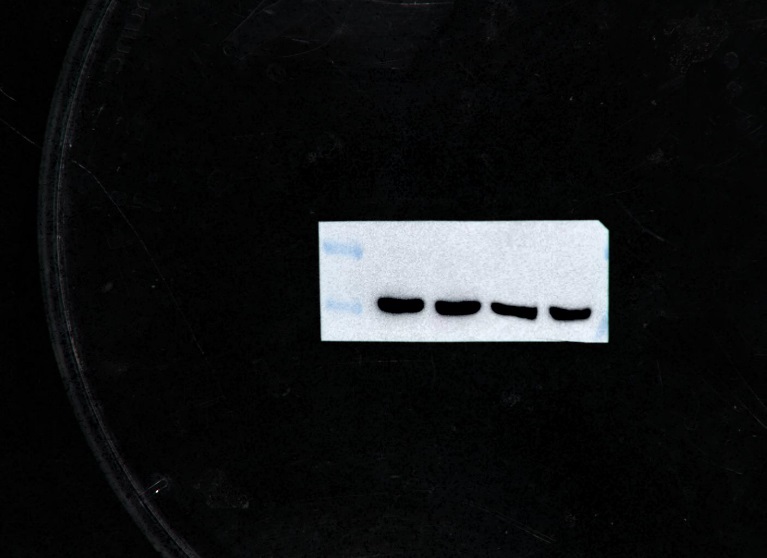 |

**Fig S4: Original Western images used for preparing Figure 9.**

| Flag | 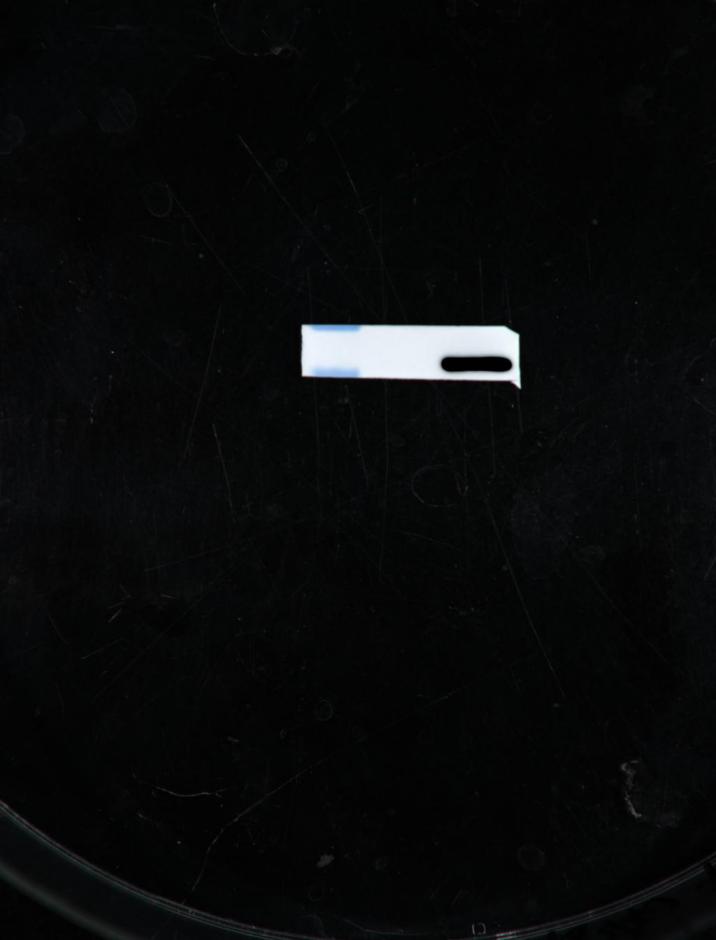 |
| --- | --- |
| ACTIN | 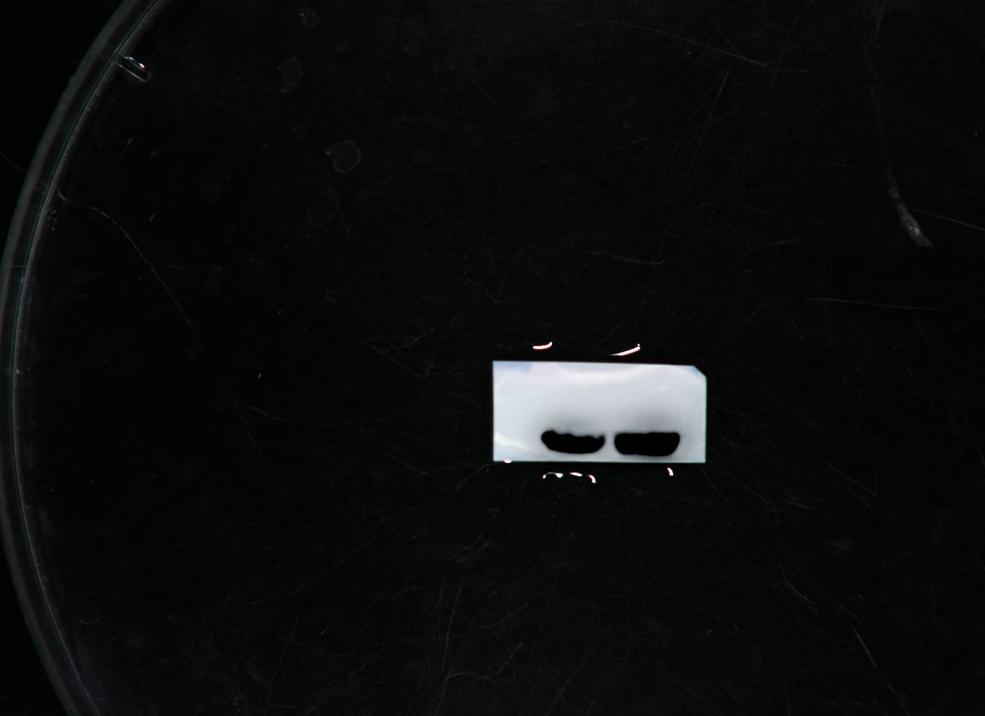 |
| Flag | 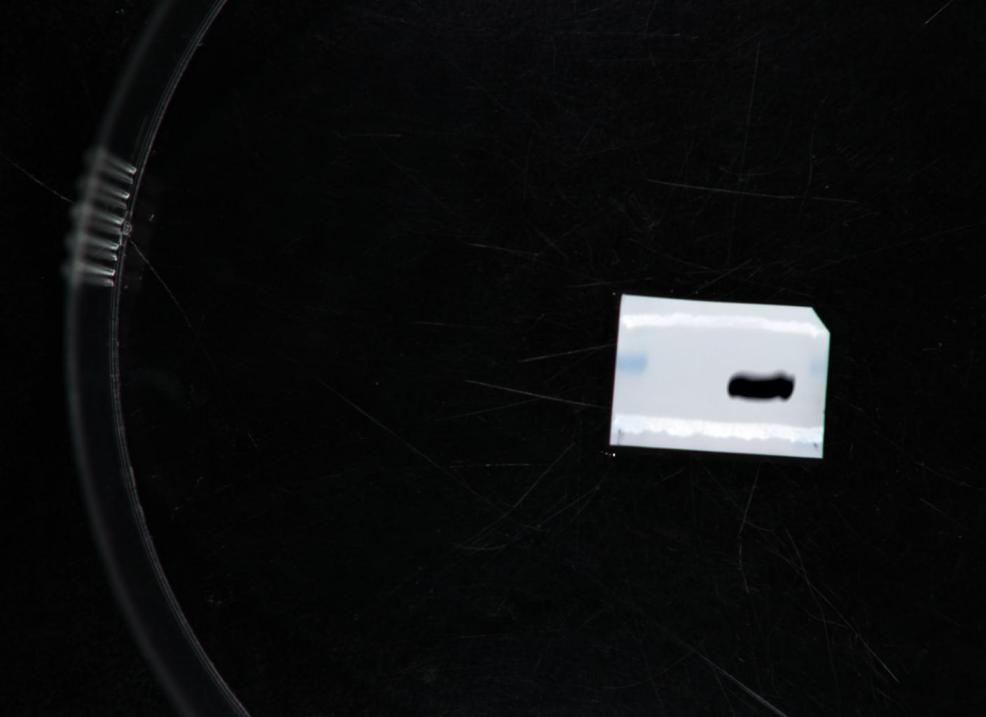 |
| ACTIN | 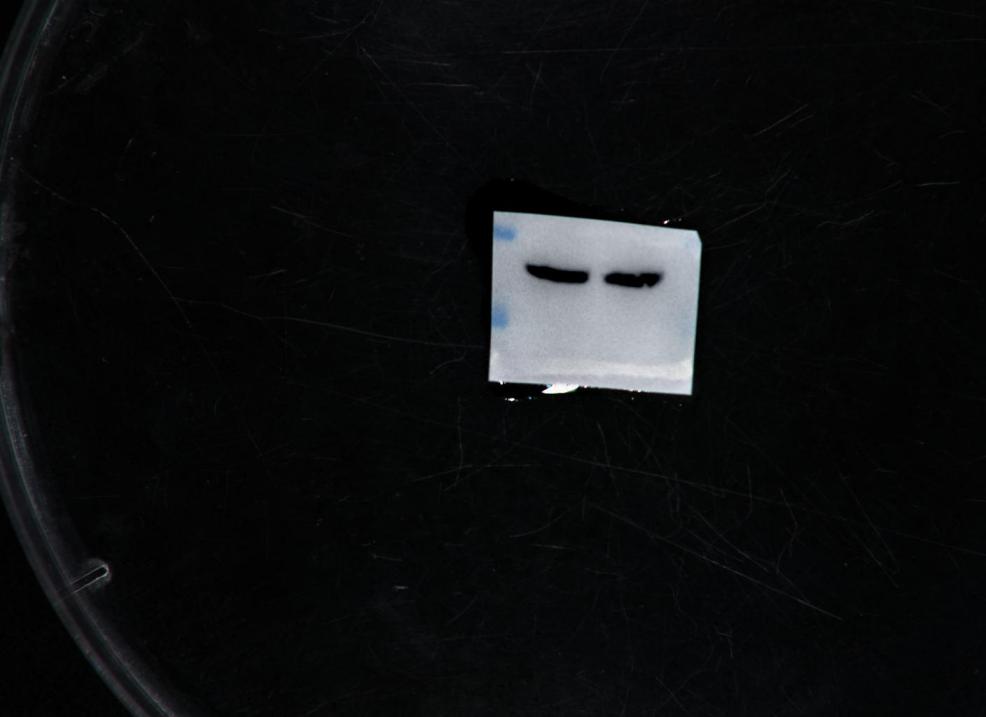 |
| Flag | 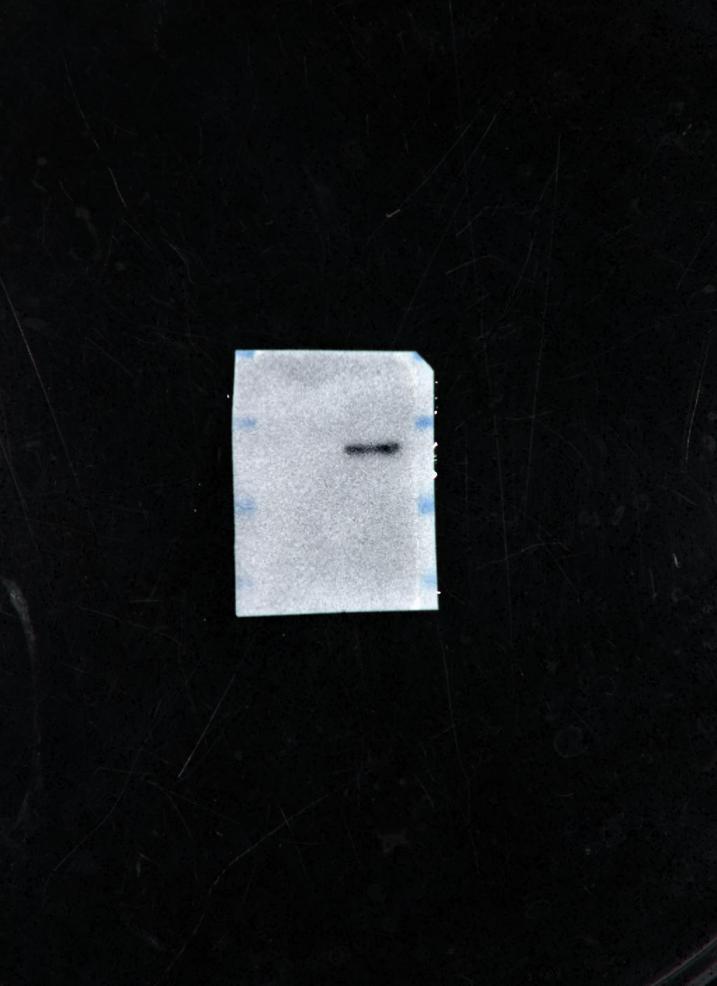 |
| ACTIN | 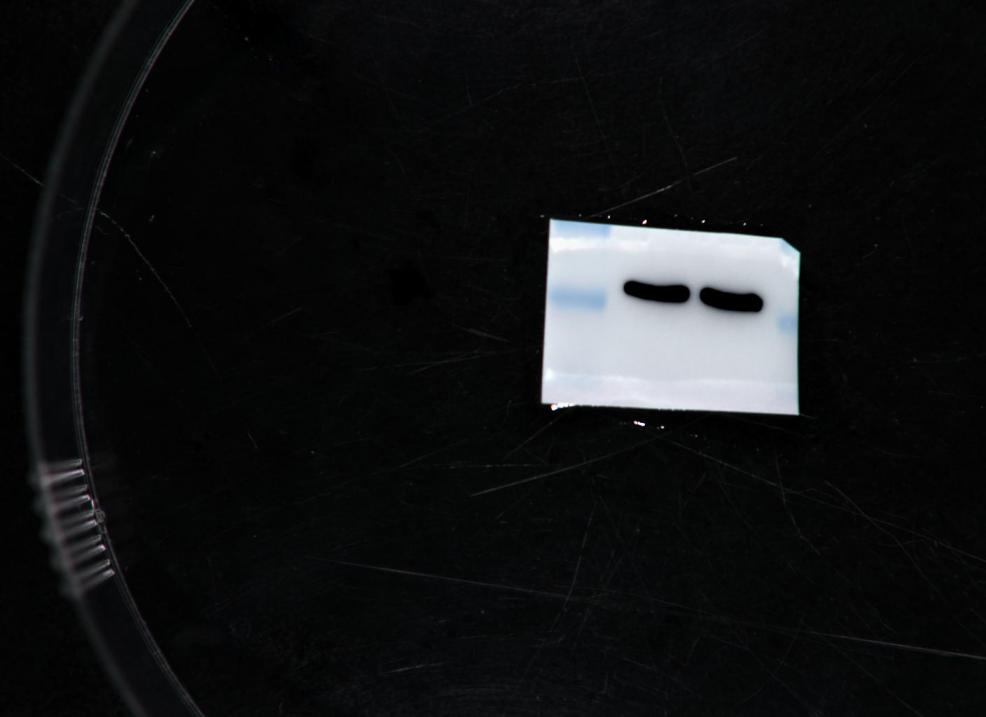 |
