# Supplementary material for: Differential Transcriptomics Analysis of IPEC-J2 Cells Single or Coinfected With Porcine Epidemic Diarrhea Virus and Transmissible Gastroenteritis Virus
Source: Front Immunol. 2022 Mar 25;13:844657. doi: 10.3389/fimmu.2022.844657 (PMC8989846; doi:10.3389/fimmu.2022.844657)
Supplement: Supplementary file 6 [file Table_1.doc]

**Supplemental Table 1 Primers and siRNAs used in this study.**

| Primers or siRNAs | Sequence（5’-3’） |
| --- | --- |
| PEDV-N-F | ctgggtgtttccgtgtacga |
| PEDV-N-R | tgccacagagcgaccattag |
| TGEV-N-F | cgaaactgcacggacgtttt |
| TGEV-N-R | ggttcttgtacgggcagcta |
| IRF1-F | tggcagcacatactggtgag |
| IRF1-R | agtgggcctgcatatggaac |
| IRF7-F | tggcagcacatactggtgag |
| IRF7-R | agtgggcctgcatatggaac |
| IFITM1-F | gatcaagagccagcacgaga |
| IFITM1-R | gcagcaccagttcaggaaga |
| IFITM3-F | ctggtccctgttcaacaccc |
| IFITM3-R | tgcaaacgatgatgaacgcaa |
| si-ssc-IFITM3_001 | gggcctccttctgatcatt |
| si-ssc-IFITM3_002 | gcagttctgcagctcataa |
| si-ssc-IFITM3_003 | gcgttcatcatcgtttgca |
| si-csa-IFITM3_001 | ccgtcaagtgcctgaatat |
| si-csa-IFITM3_002 | gctcatcgtcatccctgta |
| si-csa-IFITM3_003 | catccaccgtgatccacgt |
| sIFITM3-F | ggaattcgccaccatggattacaaggatgacgacgataagaactgcgcttcccagccct |
| sIFITM3-R | ccctcgagctagtagcctctgtaatcctttatg |
| sIFITM3-F2 | gaattcatgaactgcgcttcccagcccttc |
| sIFITM3-R2 | ctcgaggtagcctctgtaatcctttatg |
